# Randomised Trial of Text Messaging on Adherence to Cardiovascular Preventive Treatment (INTERACT Trial)

**DOI:** 10.1371/journal.pone.0114268

**Published:** 2014-12-05

**Authors:** David S. Wald, Jonathan P. Bestwick, Lewis Raiman, Rebecca Brendell, Nicholas J. Wald

**Affiliations:** Wolfson Institute of Preventive Medicine, Queen Mary University of London, Charterhouse Square, London EC1M 6BQ, United Kingdom; The National Institute for Health Innovation, New Zealand

## Abstract

**Background:**

About one third of patients prescribed blood pressure or lipid-lowering drugs for the prevention of coronary heart disease and stroke do not take their medication as prescribed. We conducted a randomized trial to evaluate text messaging as a means of improving adherence to cardiovascular disease preventive treatment.

**Methods:**

303 patients taking blood pressure and/or lipid-lowering medications were randomly assigned to being sent text messages (Text group, 151) or not being sent them (No text group, 152). Texts were sent daily for 2 weeks, alternate days for 2 weeks and weekly thereafter for 22 weeks (6 months overall), using an automated computer programme. Patients were asked to respond on whether they had taken their medication, whether the text reminded them to do so if they had forgotten, and if they had not taken their medication to determine if there was a reason for not doing so. At 6 months, use of medication was assessed.

**Results:**

Two patients were lost to follow-up, providing data on 301 for analysis. In the No text group 38/151 (25%) took less than 80% of the prescribed regimen (ie. stopped medication completely or took it on fewer than 22 of the last 28 days of follow-up) compared to 14/150 patients (9%) in the Text group – an improvement in adherence affecting 16 per 100 patients (95% CI 7 to 24), p<0.001. The texts reminded 98/151 patients (65%) to take medication on at least one occasion and lead to 20/151 (13%) who stopped taking medication because of concern over efficacy or side-effects, resuming treatment.

**Conclusions:**

In patients taking blood pressure or lipid-lowering treatment for the prevention of cardiovascular disease, text messaging improved medication adherence compared with no text messaging.

**Trial Registration:**

Controlled-Trials.com ISRCTN74757601

## Introduction

Coronary heart disease and stroke are the leading causes of death and disability worldwide. [1] Combination drug therapy with blood pressure and lipid-lowering treatment is effective in preventing cardiovascular disease events, reducing risk by about 75% with complete adherence to treatment [2],[3].

Failure to take medication is common and reduces its potential benefit, both through stopping medication completely and taking it less frequently than prescribed. It has been estimated that about one third of patients do not adhere to blood pressure or lipid-lowering treatment prescribed following a myocardial infarction or stroke (secondary prevention) and about half do not adhere to preventive treatment prescribed to prevent a first cardiovascular disease event (primary prevention) [4], [5].

Over 90% of the UK and US population own a mobile phone [6], [7], and text messaging is increasingly used in medical practice to remind patients of clinic appointments, to report test results and to adjust treatment dose [8]–[11]. Text messaging may be a useful way of reminding patients to take their medication, identifying who has not done so and the cause for not doing so if there is a reason and in such cases providing appropriate advice.

### Trial aim

We conducted a randomised trial to assess the value of text messaging as a means of improving medication adherence in patients receiving blood pressure and/or lipid-lowering treatment for the prevention of cardiovascular disease.

## Methods

The protocol for this trial and CONSORT checklist are available as supporting information; see Checklist S1 and Protocol S1.

### Eligibility and recruitment

Between February 2012 and August 2013, at 7 primary care practices in London, we enrolled 303 patients who owned a mobile telephone with text message capability and who had been prescribed blood pressure and/or lipid-lowering medication. Participant follow-up was completed in March 2014. Eligible participants were identified from electronic lists of patients who were prescribed such treatment and had a mobile phone number on record. A text message was sent to 6884 patients to introduce them to the trial and to ask them to send a text reply if they were interested in meeting an investigator with a view to participating in the trial. A further 120 patients were invited to participate when attending their primary care practice.

### Randomisation

After giving written consent, patients were randomly assigned to receive text messages (Text group) or to not receive text messages (No text group). The randomisation schedule was computer generated in blocks of 4 and allocated centrally from the coordinating centre by telephone. All other decisions regarding usual medical care were left to the discretion of the responsible physicians.

### Text messaging

The text messages were automatically generated using a software application designed for the trial, comprising three linked components; a third party text message provider, a database to record sent and received texts, and a user interface to set up a participant profile. The text messages were customized so each patient received the text just after the time they were advised to take their medication. Patients with similar requirements (text policies) were grouped together to simplify administering the texts.

Patients in the Text group were sent daily texts for two weeks, then alternate day texts for two weeks and then weekly texts for 22 weeks (6 months overall) and participants were asked to send a text message reply to each message sent, indicating whether they had taken their medication, whether the message had reminded them to take it if they had forgotten or whether they had simply not taken it. The text schedule was sent by computer and responses automatically filed, so patients who had not taken their medication or not responded could be identified and telephoned to determine whether there was a reason for not taking their medication, and if so, discussing this with a view to resolving the issue. Further details of the software application are available at www.wolfson.qmul.ac.uk/current-projects/INTERACT-trial.

### Trial outcomes

The primary trial outcome in this analysis was medication use at 6 months, exceeding 80% of the prescribed regimen. Medication use was usually determined by personal enquiry at clinic visits, or failing that, using general practice electronic prescription records. Patients were asked whether they had stopped taking their medication and if not, the number of days in the previous 28 days that medication had been missed. For patients who could not be contacted but had electronic records, the number of days missed was imputed in proportion to the number in those who were contacted. Secondary outcomes were (i) the proportion of patients continuing their medication regardless of the number of days missed and (ii) among those continuing, the proportion taking >80% of their prescribed regimen. Blood pressure measurements were taken at 6 months in patients on blood pressure lowering medication at randomisation and similarly, serum cholesterol (total and LDL) in patients on cholesterol lowering medication at randomisation.

### Sample size

We estimated that 300 patients would achieve at least an 85% power to detect a 15 percentage point or greater improvement in the proportion of patients who continued their medication and took it for at least 22 of the previous 28 days at a 5% level of statistical significance, assuming an adherence rate of 70% in the control group [4] and 20% loss-to-follow up.

### Statistical analyses

Comparisons between groups were performed using chi-squared tests for categorical variables, t-tests for continuous variables with Gaussian distributions and Wilcoxon rank-sum tests for continuous variables with non-gaussian distributions. Percentage differences in the proportion of patients who continued their medication and took it at least 22 of the last 28 days, and 95% Confidence Intervals were calculated. Subgroup analyses on the use of medication were performed according to age, sex, smoking, diabetes and history of cardiovascular disease (myocardial infarction, stroke or coronary revascularisation). The effects of text messages in these subgroups were compared using interaction tests on the basis of a significance level of 0.01 or less. Analyses were performed using Stata, version 12.

### Research ethics committee approval

The study was approved by the North Central London Research Ethics Committee in January 2011 and was registered on the ISRCTN Register (ISRCTN74757601) in April 2013 after recruitment began, due to an oversight. No related trials using this intervention are in progress and future ones will be registered.

## Results

**Figure 1:**
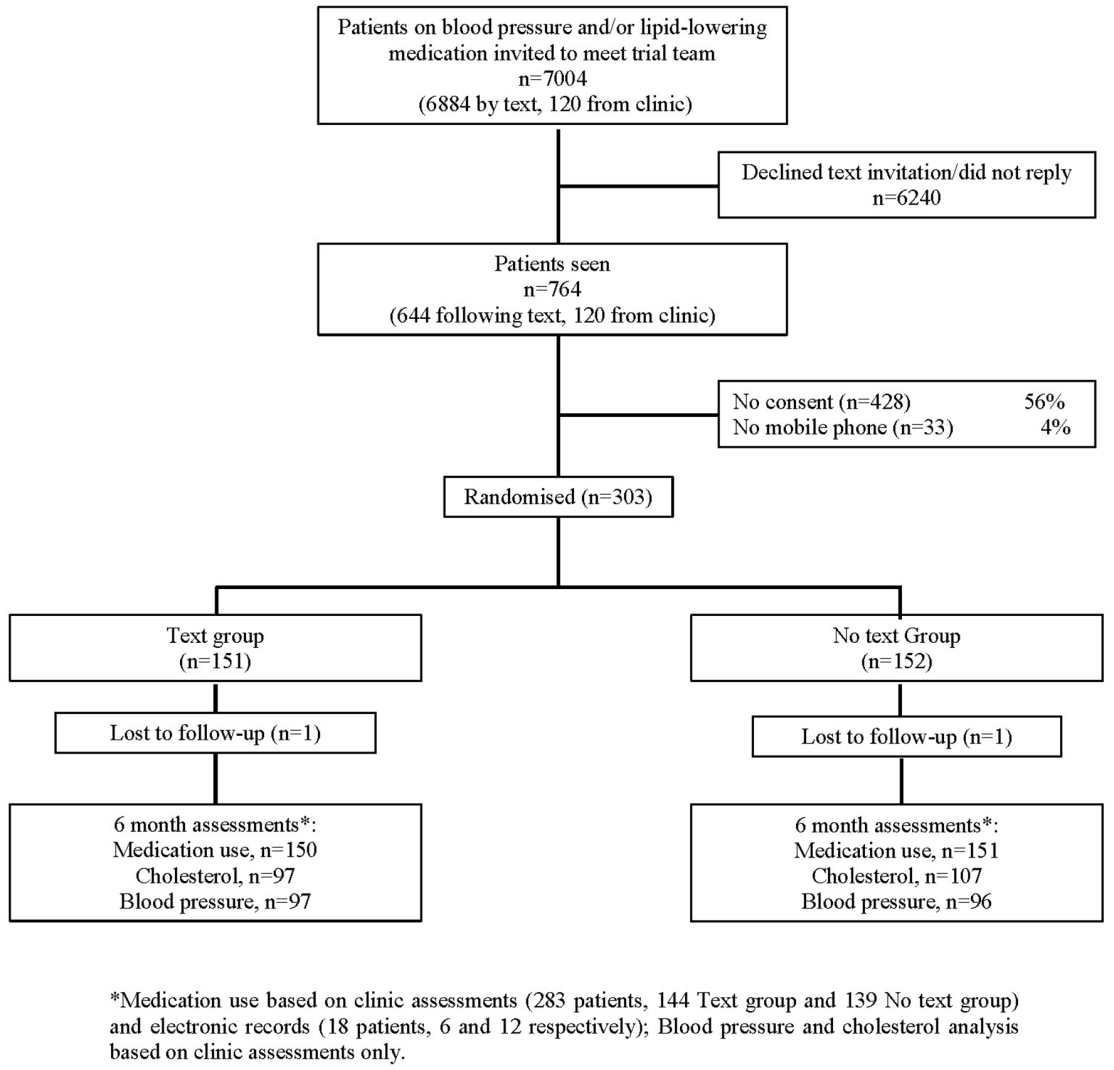
Trial Flow Diagram.

Figure 1 shows a flow diagram of the trial. Of the 303 patients enrolled, 2 were lost to follow-up providing 301 patients for analysis. The characteristics of the patients at entry to the trial were similar in the two groups (Table 1), as was the use of lipid-lowering drugs, blood pressure lowering drugs or both. The baseline characteristics of the two randomised groups were similar and no differences were statistically different.

**Table 1:** Baseline characteristics of the trial participants.

	Text (n = 150)		No Text (n = 151)	
Median age (interquartile range)	60	(54–68)	61	(49–69)
Male (%)	82	(55)	81	(54)
Smoker (%)	24	(16)	27	(18)
Medical history				
Diabetes (%)	44	(29)	55	(36)
Myocardial Infarction (%)	15	(10)	14	(9)
Stroke (%)	10	(7)	10	(7)
CABG (%)	6	(4)	5	(3)
Angioplasty (%)	14	(9)	17	(11)
Drugs				
Cholesterol lowering (%)	102	(68)	117	(77)
Statin (%)	101	(67)	115	(76)
Fibrate (%)	1	(1)	3	(2)
Blood pressure lowering (%)	102	(68)	101	(67)
ACE inhibitor (%)	51	(34)	59	(39)
ARB (%)	11	(7)	20	(13)
CCB (%)	55	(37)	60	(40)
Beta blocker (%)	19	(13)	22	(15)
Alpha blocker (%)	6	(4)	9	(6)
Diuretic (%)	33	(23)	34	(23)
Cholesterol and BP lowering (%)	54	(36)	67	(44)

ACE - Angiotensin converting enzyme, ARB - Angiotensin Receptor Blocker, CCB – Calcium channel blocker

Six months after randomisation, 14 out of 150 patients (9%) assigned to the Text group and 38 out of 151 patients (25%) assigned to the No text group stopped their medication or continued to take <80% of the prescribed regimen (took medication on fewer than 22 of the last 28 days of follow-up) – a 16 percentage point improvement in medication adherence (95% CI 7%–24%), p<0.001 (Table 2). The differences between the two trial groups, remained statistically significant when the proportion of patients who stopped medication completely and continued to take <80% of the prescribed regimen were considered separately (9% (3%–14%), p = 0.002 and 7% (0.2%–14%), p = 0.045, respectively. There were no statistically significant differences in blood pressure (132 mmHg versus 137 mmHg systolic, 77 mmHg versus 81 mmHg diastolic) or serum cholesterol (4.20 mmol/L versus 4.21 mmol/L total cholesterol and 2.29 mmol/L versus 2.22 mmol/L LDL cholesterol) between the Text and No text groups respectively.

**Table 2:** Use of medication at 6 months according to trial group.

Use of medication after 6 months	Text group (n = 150)		No Text group (n = 151)		Difference (% and 95% CI)		p-value
Stopped	3	(2%)	16	(11%)	13	(9%, (3%–14%))	0.002
Continued*							
for <22 of the last 28 days	11	(7%)	22	(14%)	11	(7%, (0.2%–14%))	0.045
Stopped or continued							
for <22 of the last 28 days	14	(9%)	38	(25%)	24	(16%, (7%–24%))	<0.001

*imputed data for 1 patient in Text group (11 v 10 observed) and 3 in the No text group (22 v 19 observed) for patients who could not be contacted.

In the Text group, 98/150 (65%) of patients were reminded to take their medication on at least one occasion and 23/150 (15%) did not take their medication on at least one occasion, because of uncertainty over the need for treatment, concern over side-effects or another medical illness that led to medication being discontinued. Following a telephone consultation 20 or these patients (13%) resumed their medication and 3 stopped completely.

The results were not materially affected by age, sex, smoking, presence or absence of diabetes and history or no history of cardiovascular disease; no statistically significant interactions were found, but the power to detect differential effects was limited.

## Discussion

The results of this trial show that in patients prescribed blood pressure and/or lipid-lowering medication for the prevention of cardiovascular disease, text messages improved the use of prescribed medication at 6 months compared with the use in patients who were not sent text messages. Stopping medication completely and taking less than 80% of the prescribed regimen were both reduced, by 9% and 7% respectively – an overall improvement in medication adherence of 16%.

The 80% cut-off (>22 of the last 28 days medication was taken) that classified a person as adhering to their medication was arbitrary, but has been used in previous publications. [4], [11] Had we adopted a lower or higher cut-off, say 75% or 85%, the difference in adherence between the text and no text groups would have been 15% or 20% respectively. The estimate is therefore reasonably robust.

The software used in this trial automated the sending of texts and recording of replies in a way that reminded patients to take their medication (about two-thirds of patients) and identified those who did not (1 in 6 patients), so the reason for not doing so could be determined and advice provided. This approach differs from previous trials which used one-way text messages to encourage the use of sunscreen [12], malaria prophylaxis [13], antibiotics [14] and blood pressure drugs [15] and did not show statistically significant improvements in adherence. Two randomised trials that, like ours, sent a text message and required a response, did show statistically significant improvements in adherence in the use of anti-retroviral treatment among HIV patients, in one trial [16], and vitamin C in healthy people in another [17]. Bidirectional texting may be an important factor in achieving a benefit.

The text messages were customized to the time of day that patients took their medication but the frequency that texts were sent was not automatically adjusted in the light of the response. Future versions of the software could be made more “intelligent” to enhance the customisation. The method was simple, is scalable and could be extended to other disorders for which drugs are taken long-term, such as asthma, epilepsy, HIV infection and multiple sclerosis.

In this trial, medication use was high because the main method of recruitment involved responding to a text invitation (response rate of 9%). The patient sample was likely to be a particularly conscientious group that was likely to adhere to their medication more than patients in general. This limited the trial's power to show statistically significant differences in cholesterol and blood pressure between the randomised groups. Such selection is probably inevitable in trials such as this, which require the consent and cooperation of the participants involved. As a consequence the results of this trial may underestimate the routine use of texting as a means of improving adherence to medication.

Several questions remain. For example, would the benefit of text messaging be improved or diminished in patients who were starting drug treatment, compared to patients who had been taking medication for some years already, as we studied here? Would the text intervention provide similar benefits in patients taking medication for other chronic disorders, such as tuberculosis or HIV infection? Would the improvement in medication adherence, observed at 6 months, be sustained if the text intervention stopped? Further research would be needed to answer these questions.

In conclusion, in this randomised trial, we found that among patients taking blood pressure and/or lipid-lowering treatment for the prevention of cardiovascular disease events, text messaging, improved the extent to which patients took their prescribed medication.

## Supporting Information

Checklist S1 **CONSORT Checklist.**
(DOC)

Protocol S1 **Trial Protocol.**
(DOC)
