# Supplementary material for: Randomised Trial of Text Messaging on Adherence to Cardiovascular Preventive Treatment (INTERACT Trial)
Source: PLoS One. 2014 Dec 5;9(12):e114268. doi: 10.1371/journal.pone.0114268 (PMC4257733; doi:10.1371/journal.pone.0114268)
Supplement: Protocol S1 — Trial Protocol. (DOC) [file pone.0114268.s002.doc]

**INTERACT Study**

**Investigation of Text Reminders on Adherence to Cardiac Treatment**

**PROTOCOL**

Version 3 01 May 2013

Study Centre

Wolfson Institute of Preventive Medicine

Queen Mary & Westfield College, University of London

(The Barts and The London

Queen Mary’s School of Medicine & Dentistry)

Charterhouse Square

London EC1M 6BQ

Representative of the Sponsor:

**Gerry Leonard**

**Head of Resources**

Joint R&D Office

5 Walden Street

London

E1 2EF

Phone: 020 7882 7260

Email: [gerry.leonard@bartsandthelondon.nhs.uk](mailto:gerry.leonard@bartsandthelondon.nhs.uk)

**Signature Page**

The investigators on the clinical study as detailed within this research protocol **(Version 1, dated 10 November)** include

**Investigator: Dr David Wald**

**Signature:**
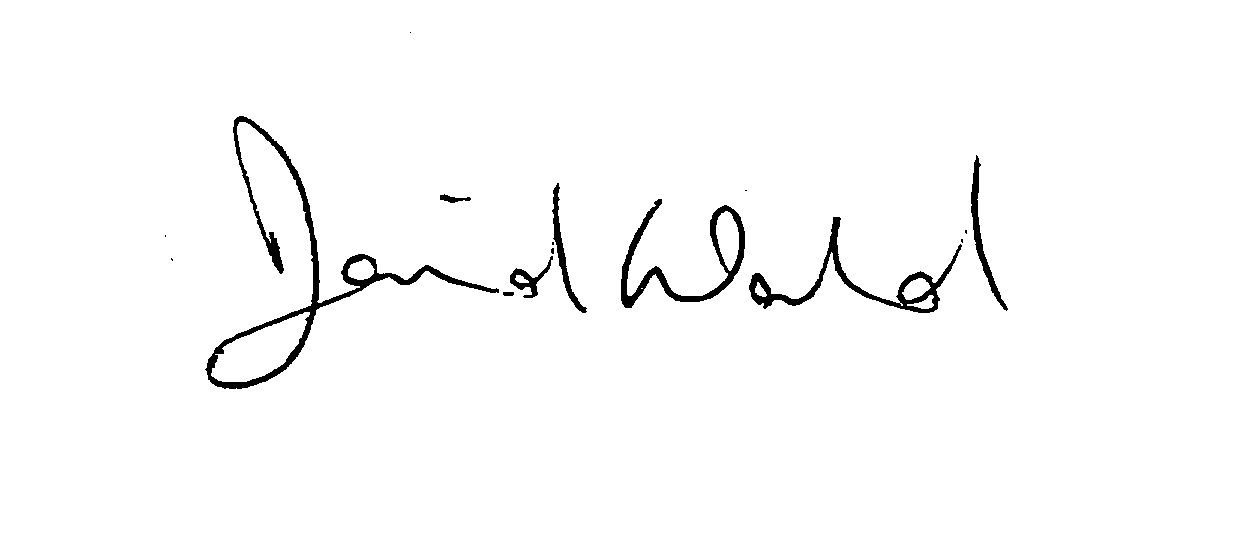
 **Date: 10 Nov 2010**

**Site:** Wolfson Institute of Preventive Medicine, Barts and the London School of Medicine, Queen Mary & Westfield College, University of London

| **TITLE** | **Investigation of Text Reminders on Adherence to Cardiac Treatment** |
| --- | --- |
| **SHORT TITLE** | INTERACT Study |
| **Protocol Version Number and Date** | Version 3 [ May 01 2012] |
| **Methodology** | Randomised controlled trial |
| **Study Duration** | 18 months |
| **Co-ordinating Centre** | **Wolfson Institute of Preventive Medicine** |
| **Objectives** | To assess the effect of programmed text message reminders on adherence to cardiovascular disease preventive medication. |
| **Number of Subjects/Patients** | 300 |
| **Main Inclusion Criteria** | Patients prescribed lipid lowering and/or blood pressure lowering medication. |
| **Intervention** | “Text Message Programme (TMP)”: periodic text messages, adapted in frequency to the responses received coupled with telephone or clinic consultation to resolve non-adherence. |
| **Outcome Measures** | **Primary**: Non-adherence to combination therapy  **Secondary**: serum cholesterol, blood pressure and cost-effectiveness |
| **Statistical Methodology and Analysis** | To estimate the absolute and relative differences (and 95% confidence intervals) in non-adherence to preventive therapy in the intervention and control arms, 6 months after enrolment in the study. Based on 30% non-adherence rate in the control arm and a 20% loss-to-follow up, a study of 300 participants has 80% power to detect a 50% reduction in non-adherence from TMP at the 5% level of significance. |

**Index Page Number**

1. **Introduction 6**
   1. Abstract 6

1.2 Background 6

**2.** **Study Aims and Design of Proposed Investigation 7**

2.1 Research Aim 8

2.2 Study Objectives 8

2.2 Study Design 8

2.3 Flow Diagram of study 8

**3. Study Population 10**

- 1. Inclusion Criteria 10
  2. Exclusion Criteria 10

**4. Study Procedures 10**

- 1. Recruitment 11
  2. Consent 11
  3. Data collection 11
  4. Randomisation 11
  5. Clinical and Laboratory Assessments 12
  6. Schedule of Assessment 13

**5. Study Endpoints 13**

5.1 Primary endpoint 13

5.2 Secondary endpoints 14

**6. Statistical Considerations 14**

6.1 Number of Participants 14

6.2 Data Analysis 14

6.3 Data access and security 15

**7. Data Handling & Record Keeping 15**

7.1 Confidentiality 15

7.2 Case Report Form 16

7.3 Record Retention and Archiving 16

7.4 Compliance 16

7.5 Clinical Governance Issues - Ethical Considerations 16

**8. Study Committees 16**

**9. Publication Policy 16**

**10. Insurance to Cover Indemnity for Non-Negligent Harm 16**

**11. References 18**

**Index Page Number**

**Appendix A letter to uncontactable Patients 19**

**Appendix B GP Letter 20**

**Appendix C Consent Form 21**

**Appendix D Information leaflet 22**

**Appendix E Text 23**

**Appendix F Letter 24**

**1. INTRODUCTION**

***1.1 Abstract***

A randomised controlled trial of text message reminders will be conducted in 300 patients who are receiving combination therapy with at least one lipid lowering drug (eg. a statin) and/or one blood pressure lowering drug (eg. an ACE-inhibitor). The intervention arm will consist of a “Text Message Programme” – periodic text messages asking whether patients have taken their medication, adapted, over time in their frequency, to the responses received, coupled with telephone or clinic follow-up to resolve reasons for non-adherence. The control group will receive standard care. Non-adherence to therapy (discontinuing one or more of the starting combination) will be assessed at 6 months by questionnaire. Serum cholesterol and blood pressure measurement will be undertaken as independent, objective measures of drug use. Allowing for a 20% loss-to follow up, the study has 80% power to identify a statistically significant reduction in non-adherence of 50% in the intervention group at a 0.05 level of statistical significance.

***1.2 Background***

Coronary heart disease (CHD) is the leading cause of death in both developed and developing countries.^1^ Approximately one third of all deaths are due to CHD in England and Wales.^2^

Combination drug therapy with antiplatelet, blood pressure and serum cholesterol lowering drugs is effective in reducing the risk of first cardiovascular disease events; about ¾ of all heart attacks and strokes could be prevented with complete adherence to treatment.

Non-adherence to medications is however common for patients with cardiovascular diseases and greatly reduces the preventive effect. Jackevicius and colleagues found that about one quarter of all patients with a history of myocardial infarction stopped their cardiac medications within 1 week of discharge from hospital^3^ and more than half had discontinued statin therapy two years later.^4^ Newby and colleagues found that less than half of patients with angiographically confirmed coronary artery disease remained on statins one year after starting treatment in hospital (44% non-adherence) and 79% of patients who had been prescribed the three required classes of preventive therapy (antiplatelet therapy, lipid lowering therapy and blood pressure lowering therapy) stopped at least one class of drug after one year.^5^ The levels of non-adherence may be under-estimated in these studies, based as they were on prescription re-fills and self-reported continuation on treatment, rather than objective measures (blood or urine tests) of drug intake.

Non-adherence to medical treatment is also a major health-economic problem. An estimated £100 million is wasted each year in Britain because of unused prescribed medications^6^. Reasons for non-adherence include side-effects of treatment, a belief that treatment is not-required, not remembering to take treatment, loss of interest in prevention when treatment has no perceptible benefit, and loss of contact with the prescribing medical team over time^7^. A recent publication from the National Institute for Health and Clinical Excellence called for interventions to improve the extent to which patients remain on effective medication for chronic conditions such as coronary heart disease^8^, which cost the NHS about £500 million in 2009.^9^ Non-adherence to cardiovascular medication therefore has a personal and economic impact.

Mobile telephone technology is increasingly used in clinical practice to improve contact with patients. Text message reminders are used to remind patients of clinic appointments^10^ and the need to attend for immunisation^11^. Text messaging has also been used to report blood pressure readings, blood results in patients with HIV and alter dose of medication in patients with epilepsy receiving anti-convulsant therapy^12^. The use of text messaging lends itself to improving adherence to take long-term medication, both by helping to establish a routine through text message reminders and also by identifying and correcting the causes of non-adherence as they arise. There is however a lack of randomised trial evidence demonstrating medical and cost-effectiveness of this approach. One randomised study on 70 participants in Australia evaluated the effectiveness of text message reminders on adherence to sunscreen application and showed a 92% increase in the use of sunscreen in the intervention arm^13^. There are no randomised studies of the use of text-message technology on adherence to cardiovascular preventive medication.

**2. Study Aims and Design of Proposed Investigation**

**2.1 Research Aim**

To conduct a randomised trial to assess the effect of a Text Message Programme (TMP) on non-adherence to combination cardiovascular disease preventive medication. The TMP involves sending text messages to patients, adapted in frequency to the responses received, to encourage medication adherence and to identify patients not-adhering to medication so that telephone or clinic-based assistance can be provided to resolve causes of non-adherence..

**The TMP is therefore, not only a means of reminding patients to take their medication, it is also a method for identifying those patients who, for whatever reason, have discontinued their treatment, so that medical assistance can be quickly offered to resolve the problem.**

**2.2 Study Objectives**

**Primary Objective**: to determine, in each randomised group, non-adherence to combined treatment with lipid lowering and/or blood pressure lowering treatment (Combination Therapy).

**Secondary Objectives**:

(i) Serum cholesterol and blood pressure levels at the end of follow up.

(iii) Cost-effectiveness of the Text Message Programme

***2.3 Study Design***

The study will be conducted in two parts:

Part 1: A pilot study on 30-50 patients to assess the feasibility of the text message protocol, the recruitment rate and therefore the number of recruiting centres required. Appendix A lists the questions that will be asked.

Part 2: Recruitment of a total of 300 patients.

The study will adopt a parallel group design

***2.4 Flow Diagram of Study***

Flow diagram based on:

**150**

Standard Care* alone

**150**

**45**

Text Messaging Program plus standard care*

**Non-adherence**

**23**

**6 month**

Self-reported adherence

Serum cholesterol and BP

**300 patients prescribed, lipid lowering and/or BP lowering treatment**

1. Non-adherence in control arm of 30% (ie 45 out of 150)
2. Expected 50% reduction in non-adherence by TMP
3. Loss to follow-up of 20%

*Standard care includes, hospital clinic review, General Practitioner consultations and community pharmacist review.

The figure bellow is indicative of the text message program protocol that will be adopted.

***Text Message Program Algorithm***

**Randomisation**

Text message sent **DAILY**

Text message sent on

**ATERNATE DAYS**

Text message sent **WEEKLY**

Follow up visit

**Phase 1**

**Phase 2**

**Phase 3**

**Week 1-2**

**Week 3-4**

**Weeks 5+**

**End of study**

Patients receive a daily text message for the first 2 weeks of the study (*Phase 1*), every alternate day for the second 2 weeks of the study (*Phase 2*), and weekly thereafter (*Phase 3*).

The wording of the text messages will vary according to the phase of the TMP protocol. An example of the daily text massage reminder is:

‘***Hello Mr/Mrs……. Have you taken your heart medication today? Please text back “Y” if the answer is Yes, I took my medication before receiving this text”, “P” if the answer is “Yes, the message prompted me to take my medication” and “N” if the answer is “No”. Thank you. From The Barts and The London Heart Team***’.

Any non-responders or patients who reply back ‘NO’ in response to the message will receive a telephone call form the study coordinator, to determine whether the patient is experiencing problems taking their medication, and resolve this if possible (e.g. the patient has run out of medication or they have been abroad).

If specialist advice is needed (e.g. whether symptoms may be a side-effect of treatment) participants will be informed that they will be contacted by a study pharmacist or doctor within the next few days. If the problem can not be resolved over the phone the patient will be advised to make an appointment with their GP. A letter will be sent to the patient’s GP summarising the problem with their medication and a copy of this letter will be sent to the patient’s home address.

If patients can not be contacted by telephone, a letter will be sent to their home address informing them that we have been unable to contact them by telephone and asking them to contact the study team (Appendix B).

***Follow-up Visit***

6 months from randomization, patients will be asked to return to attend a clinic follow-up visit. Each Patient will be asked questions to assess their adherence to lipid lowering and/or blood pressure lowering medication.

Other follow up measurements will include: Serum cholesterol, and blood pressure.

If a clear difference in non-adherence between the intervention and control arms is observed after 6 months, a further follow-up visit after 18 months, would be arranged to determine whether the effect of the TMP is sustained.

**3. STUDY POPULATION**

***3.1 Inclusion Criteria***

Participants will be men and women of all ages who own a mobile phone with text message capability. Patients will be on a combination of medicines (at least a lipid lowering drug and/or a blood pressure lowering drug) for cardiovascular disease preventive treatment.

It is anticipated that most patients will be on both classes of drug (since these are recommended for cardiovascular disease prevention). However patients who for some reason are only on one class (eg. they may be intolerant of all blood pressure lowering drugs) will still be eligible and non-adherence to the one class will be assessed.

***3.2 Exclusion Criteria***

There are no exclusions

**STUDY PROCEDURES**

***4.1 Recruitment***

Participants will be recruited from angiography and cardiovascular disease prevention clinics (eg. cardiology, hypertension and diabetes clinics). Patients who are on appropriate cardiovascular preventive treatment will be approached by a study investigator in the clinic waiting area and given a study information leaflet. After patients have been seen by the doctor they will be seen again by the study investigator who will answer any questions they may have about the study and ask them if they would be willing to participate. Written consent will then be sought. Patients on appropriate treatment could also be identified by the clinical care team from a search of the practice database and sent a text message (Appendix E) or a letter (Appendix F) informing them of the study and asking them to contact the study team if they would like to find out more about the study. Interested participants would then be offered an appointment to meet a study investigator at their local general practice, to answer any questions they may have and ask them if they would be willing to participate. Written consent will then be sought.

***4.2 Consent***

All patients will be asked to give written consent prior to participation as described above.

***4.3. Data Collection***

When a person agrees to participate the following will be recorded: Participant’s name, age, gender, address, date of birth, mobile phone number, and GP details. Each participant will be allocated a unique study number.

Details of the participant’s medical history will be recorded including prior history of cardiovascular disease, diabetes, smoking and cardiovascular risk factors.

***4.4 Randomisation***

Participants will be randomly allocated to receive either the TMP (the intervention arm of the trial) or standard care (the control arm of the trial). Participants will be randomised after they have given consent to be in the trial, so that the decision to participate has no bearing on which arm of the trial they are allocated to. A random sequence will be generated by computer in blocks of 12 to ensure balanced 1:1 randomisation.

***4.5 Clinical and Laboratory Assessments***

Blood pressure measurements, blood for serum cholesterol and thomboxane estimation will be collected at the end of the study.

**Blood Pressure**

Seated blood pressure will be measured using an electronic sphygmomanometer and an appropriately sized cuff on the right arm of participants. Two readings will be taken and the mean calculated.

**Blood samples**

Serum cholesterol measurements are usually routinely available in patients on lipid lowering treatment and these will be recorded at baseline. A venous blood sample, identifiable by the participants unique study number and the time and date when the sample was taken, will be collected at the 6 month follow-up visit and spun to provide serum for analysis of total cholesterol, HDL cholesterol, triglycerides and calculated LDL cholesterol.

**Other measurements**

Each patient will be questioned at the end of the study to assess non-adherence to their combination therapy prescribed at the start of the trial. Adherence will be assessed by asking the patient the following questions:

1) Are you still taking your i i) Cholesterol lowering drug (eg. statin) ii) Blood pressure lowering drug? (eg. lisinopril)

2) In the past month were there any days when you did not take your medication?

3) If so, how many days did you not take your medication?

**Samples stored for future research**

A whole blood sample and a hair sample will be collected at the follow up visit at the end of the trial and will be kept for future analyses at the Wolfson Institute laboratory. Whilst not all analyses can be anticipated at present, these might, for example, include analysis of drug metabolites in the hair sample. Consent for this will be sought.

***4.6 Schedule of Assessment***

| **Procedure** | **Day 0^a^** | **Weeks**  **1-2** | **Weeks**  **3-4** | **Weeks**  **5+** | **Follow up** |
| --- | --- | --- | --- | --- | --- |
| Consent | x |  |  |  |  |
| Medical History | x |  |  |  |  |
| Blood Pressure^b^ | x |  |  |  | x |
| Blood sample for serum Cholesterol | x† |  |  |  | x |
| Hair sample |  |  |  |  | x |
| Text message sent daily |  | x |  |  |  |
| Text message sent every alternate day |  |  | x |  |  |
| Text message sent weekly |  |  |  | x |  |
| Text message sent monthly |  |  |  |  |  |
| Extra text message if necessary |  | x | x | x |  |
| Telephone call if necessary |  | x | x | x |  |
| Questionnaire on Adherence |  |  |  |  | x |
| *Prescription refill ascertainment |  |  |  |  | x |

a: Day 0 = Day of randomisation

b: Two blood pressure readings will be performed, and the mean recorded.

† part of routine care

**** possible additional assessment, if practical***

**5.** **STUDY ENDPOINTS**

***5.1 Primary end-point***

The primary endpoints are:

1. Continuation rate: The proportion of patients in each arm remaining on combination therapy at the end of follow-up.*
2. Number of days in the last month of the study that patients missed taking their medication.

****NB:***

***If the patient started on two classes (e.g. lipid lowering plus blood pressure lowering) and is taking only one (e.g. lipid lowering) at follow-up they would be non-adherent.***

***If the patient started on one class (eg. lipid lowering) and is taking none at follow-up they would be non-adherent.***

***If patients change drugs within the same class they would remain adherent.***

***i.e. if the starting treatment included lipid lowering (simvastatin) and blood pressure lowering (lisinopril) and the follow-up regimen included atorvastatin (lipid lowering) and losartan (blood pressure lowering), then the patient would have adhered to starting regimen.***

***5.2 Secondary endpoints***

1. Mean Serum cholesterol and blood pressure levels, in each arm at the end of follow up

2. Cost-effectiveness of the Text Message Programme

**6. STATISTICAL CONSIDERATIONS**

***6.1 Number of Participants***

The TMP is expected to reduce non-adherence by 50%. Non-adherence among patients with angiographically confirmed coronary artery disease has been reported to be at least 50%^3^. To be conservative we have taken the non-adherence rate in the control arm to be 30%. A study of 300 participants would have 80% power to detect a 50% reduction in non-adherence at the 5% level of significance. This allows for a 20% loss-to-follow up.

***6.2. Data Analysis***

A statistical analysis of the results will be undertaken at the completion of the study. The control-adjusted differences in adherence serum cholesterol and systolic and diastolic blood pressure will be computed.

Cost-effectiveness

The direct costs of administering the TMP will be recorded, including the number of text messages sent and received, the number of telephone calls made, any time spent with GP, and any clinic contact that results. The preventive effect of any improvement in adherence to combination therapy will be estimated from data on efficacy from randomised studies and the cost-effectiveness estimated in terms of the number of life-years gained as a result of the TMP.

***6.3. Data access and security***

All records will be kept securely and confidentially and analyses for reports and publications will present anonymous details. The Wolfson Institute of Preventive Medicine is part of Barts and The London Queen Mary’s School of Medicine, University of London which is registered under the Data Protection Act. In the Wolfson Institute there are two levels of security: (i) the data will be coded and kept on a network fileserver to which access is available only to a limited number of authorised users; (ii) user permissions ensure that even when logged on to the server the database is invisible to all but specifically authorised users. The server is behind a “firewall” which isolates it from the rest of the College network. It is physically protected in a locked room to which only computer staff have access, backed up every evening. Access to the building is controlled.

**7. Data Handling & Record Keeping**

***7.1 Confidentiality***

The Investigators will ensure that patient anonymity is protected and maintained. They will ensure that their identities are protected from any unauthorized parties. Information with regards to study patients will be kept confidential and managed in accordance with the Data Protection Act, NHS Caldicott Guardian, The Research Governance Framework for Health and Social Care and Research Ethics Committee Approval. All participant identifiable information will be anonymised with regard to any report or publication relating to this study.

The same standard will be followed if any patient information needs to be sent to a third party (including correspondence/communication to central laboratories, text message service administrators, or sponsor).

**7.2** ***Case Report Form***

The Study investigators will be responsible for completion of the Case Report Form for each participant which will collect information as detailed in section 5.

***7.3 Record Retention and Archiving***

All records are the responsibility of the Investigators and will be keptsecurely. When the research is complete, it is the intention that the records will be kept for a further 20 years.

***7.4 Compliance***

The Investigators will ensure that the study is conducted in compliance, the principles of GCP and the Royal College of Physicians Guidance on Research Ethics.

***7.5 Clinical Governance Issues***

*Ethical Considerations*

This protocol and any subsequent amendments, along with any accompanying material provided to the patient in addition to any advertising material will be submitted by the Investigator to an Independent Research Ethics Committee. Written Approval from the Committee will be obtained and subsequently submitted to the JRO for Final R&D approval.

**8. Study Committees**

A public engagement committee will be convened to review the results of the pilot phase of the study to determine whether any changes to the protocol or text message algorithm may be needed prior to completion of the study.

**9. Publication Policy**

It is our intention to present the results of the cross-over study at scientific meetings and publish the results in scientific journals. All data will be anonymised for this purpose.

**10. INSURANCE TO COVER INDEMNITY FOR NON-NEGLIGENT HARM**

Queen Mary (University of London) will provide indemnity for non-negligent harm and will provide indemnity for its staff for negligent harm. Compensation for any injury caused by taking part in this study, however unlikely, will be in accordance with the guidelines of the ABPI. These guidelines recommend compensation without the participant having to prove that the investigators are at fault. This applies in cases where it is likely that such injury results from the participant following this protocol. The participant’s right at law to claim compensation for injury where he or she can prove negligence is not affected.

**11. REFERENCES**

| 1 |  | Wald NJ, Law MR. A strategy to reduce cardiovascular disease by more than 80%. *BMJ* 2003;**326**:1419-23 |
| --- | --- | --- |
| 2 |  | Law MR, Wald NJ, Rudnicka AR. Quantifying effect of statins on low density lipoprotein cholesterol, ischemic heart disease and stroke: systematic review and meta-analysis. *BMJ* 2003;**326**:1423-7 |
| 3 |  | Jackevicius CA, Li P, Tu JV. Prevalnec, predictors and outcomes of primary nonadherence after acute myocardial infarction. Circulation 2008;117:1028-1036 |
| 4 |  | Jackevicius CA, Mamdani M, Tu JV. Adherence with statin therapy in elderly patients with and without acute coronary syndromes. JAMA 2002;288:462-467 |
| 5 |  | Newby LK, LaPointe NMA, Chen AY, Kramer JM, Hammill BG, DeLong ER et al. Long-term adherence to evidence based secondary prevention therapies in coronary artery disease. Circulation.2006;113:203-212 |
| 6 |  | Department of Health. Pharmacy in England, building on strengths - delivering the future. 1-141. 2008. |
| 7 |  | Ho M.P, Bryson C.L, Rumsfeld J.S. Medication Adherence. Its Importance in Cardiovascular Outcomes. Circulation 2009;119:3028-3035 |
| 8 |  | Medicines Adherence: involving patients in decisions about prescribed medicines and supporting adherence, Full Guideline January 2009 , National Collaborating Centre for Primary Care, The Royal College of General Practitioners |
| 9 |  | Prescriptions Dispensed in the community: England, Statistics for 1999 to 2009. The Health and Social Care Information Centre. Prescribing support Unit. |
| 10 |  | Leong KC, Chen WS, Leong KW, et al. The use of text messaging to improve attendance in Primary care: a randomized controlled trial. Family Practice 2006;**23**:699-705 |
| 11 |  | Vilella A, Bayas JM, Diaz MT, et al. The role of mobile phones in improving vaccination rates in travelers. Preventive Medicine 2004;**38**:503–509 |
| 12 |  | National Hospital and iP**LATO** Launch New Service to Support People with Epilepsy http://www.iplato.net/uncategorized/national-hospital-and-iplato-launch-new-service-to-support-people-with-epilepsy.html |
| 13 |  | April W et al. Text-Message Reminders to Improve Sunscreen Use: A randomized, controlled Study Using Electronic Monitoring. Arch Dermatol 2009; **145(No 11)**; 1230-1236 |

enior Lecturer & Consultant Cardiologist

**Appendix A: Letter to Uncontactable Patients**

Centre for Environmental and Preventive Medicine

| WOLFSON INSTITUTE OF PREVENTIVE MEDICINE  Director: Professor Sir Nicholas Wald FRCP FRS | Charterhouse Square London EC1M 6BQ |
| --- | --- |

Tel: +44 (0)20 7882 6269

Fax: +44 (0)20 7882 6270

E-mail: [d.s.wald@qmul.ac.uk](mailto:d.s.wald@qmul.ac.uk)

Dear Mr/ Mrs/ Miss………..

We are writing to you with regards to the INTERACT trial in which you are participating.

We have not been able to reach you by telephone or text message.

Please could you contact the study team on: **0207 882 6289** so that we can check that the contact details we hold for you are correct and discuss any problems that you may have regarding your heart medication.

Yours sincerely,

Dr David Wald

Consultant Cardiologist and Senior Lecturer

Barts and the London School of Medicine

Senior Lecturer & Consultant Cardiologist

Barts and The London

School of Medicine and Dentistry

University of London

INTERACT

Version 1, 10 November 2010

**Appendix B: GP Letter**

Centre for Environmental and Preventive Medicine

| WOLFSON INSTITUTE OF PREVENTIVE MEDICINE  Director: Professor Sir Nicholas Wald FRCP FRS | Charterhouse Square London EC1M 6BQ |
| --- | --- |

Tel: +44 (0)20 7882 6298

Fax: +44 (0)20 7882 6270

E-mail: [d.s.wald@qmul.ac.uk](mailto:d.s.wald@qmul.ac.uk)

GP Name and Address

RE: Patient Name, date of birth

Dear Dr …….,

I am writing to let you know about a randomised trial being carried out at Barts and the London School of Medicine, and to say that your patient (above) has agreed to take part.

We are examining the value of a text message programme (TMP) on reducing non-adherence to cardiovascular preventive medication among patients who are prescribed the lipid lowering drug and/or a blood pressure lowering medication.

We have made it clear that the decision to participate is entirely voluntary.

Each patient randomised to the TMP arm of the trial will receive periodic text messages to determine whether they have taken their cardiac medication. Patients who do not respond or whose response is that they have been unable to take their medication will be telephoned by a study doctor or pharmacist to explore and resolve the reason why medication is not being taken as prescribed. If the problem can not be resolved by phone (e.g. a side effect develops that requires a change in therapy) we will advise the patient to make an appointment with you at your general practice. We will write to you explaining the patient’s particular problem, and send a copy of the letter to the patient.

The TMP is therefore, not only a means of reminding patients to take their medication, it is also a method for identifying those patients who, for whatever reason, have discontinued their treatment, so that medical assistance can be quickly offered to resolve the problem.

I enclose a study information leaflet which has been given to your patient. Please do not hesitate to contact me if you wish to know anything further about the study.

Yours sincerely,

Dr David Wald

Consultant Cardiologist and Senior Lecturer

Barts and The London

School of Medicine and Dentistry

University of London

INTERACT

Version 1, 10 November 2010

**Appendix C: Consent Form**

Centre for Environmental and Preventive Medicine

| WOLFSON INSTITUTE OF PREVENTIVE MEDICINE  “Queen Mary & Westfield College, University of London” | Charterhouse Square London EC1M 6BQ |
| --- | --- |

Tel: +44 (0)20 7882 6189

Fax: +44 (0)20 7882 6270

E-mail: d.s.wald@qmul.ac.uk

Participant Identification Number:

Investigator:…………………………………………

**INTERACT Trial**

**CONSENT FORM**

**Please initial box**

| 1. I confirm that I have read the information sheet (version 1, 10 November 2010), for the above study and have had the opportunity to ask questions. |  |
| --- | --- |
|  |
| 1. I understand that my participation is voluntary and that I am free to withdraw at any time, without giving any reason, without my medical care or legal rights being affected. |  |
|  |
| 1. I understand that relevant sections of my medical notes, data collected during the study and data provided by the NHS Information Centre, may be examined by responsible individuals from regulatory authorities and/or from Barts and the London/Queen Mary University of London, where it is relevant to my taking part in the study or in future research. I give my permission for these individuals to have access to my records. |  |
|  |
| 1. I agree that my blood sample can be stored in the Wolfson Institute of Preventive Medicine for future research. |  |
|  |
| 1. I agree that my hair sample can be stored in the Wolfson Institute of Preventive Medicine for future research. | b |
|  |
| 6. I agree to take part in the above study. |  |
|  |

________________________ ________________ ____________________

Name of participant Date Signature

________________________ ________________ ____________________

Name of Investigator Date Signature

One copy for researcher, one for participant

Barts and The London

School of Medicine and Dentistry

University of London

INTERACT

Version 1, 10 November 2010

**Appendix D: Information Leaflet**

**Appendix E : Text Message**

“Your GP surgery, [Name of General Practice], is assisting colleagues from Barts and the London Hospital Medical School carry out a research project to see if Text Messages help you remember to take your pills. If you are interested in finding out more please reply YES to this message or call 02078825746

**APPENDIX F: Letter**

On Clinical Practice Letter-head

Address

Date

Dear [Insert name of patient]

Your General Practice is helping colleagues at Barts and The London Hospital medical School carry out a research project to see whether mobile phone text messages can help patients take their blood pressure and cholesterol lowering medications. We thought you may be interested in participating in the study because you are prescribed one or both of these types of treatment.

It is well recognised that it can be difficult to remember to take medication regularly and many people stop their pills when they are still needed. Mobile phone text messaging may be a practical solution.

Participation in the study is voluntary and limited to people with a mobile phone. An initial meeting at the general practice would take place to meet one of the research team who would review your suitability for the study, answer any questions you may have and seek your consent to be in the study. Participants would be allocated at random to periodically receive text messages or not to receive text messages. There would be a further meeting at you general practice after 6 months to review your medications, cholesterol and blood pressure.

If you would like to take find out more about the study please call 0207 882 5746 and one of the research team will be able to answer any questions you may have and arrange to meet you at your General Practice if you are interested in participating in the study.

With best wishes

[name of GP]
